# Blending Video Therapy and Digital Self-Help for Individuals With Suicidal Ideation: Intervention Design and a Qualitative Study Within the Development Process

**DOI:** 10.2196/49043

**Published:** 2023-09-21

**Authors:** Rebekka Büscher, Lasse B Sander, Mattis Nuding, Harald Baumeister, Tobias Teismann

**Affiliations:** 1 Medical Psychology and Medical Sociology Faculty of Medicine University of Freiburg Freiburg Germany; 2 Department of Clinical Psychology and Psychotherapy Ulm University Ulm Germany; 3 Mental Health Research and Treatment Center Ruhr University Bochum Bochum Germany

**Keywords:** blended care, digital interventions, video therapy, suicidal ideation, personalized treatment, cognitive behavioral therapy, CBT, suicide, suicidal, digital intervention, digital health, video, web-based module, blended, self-help

## Abstract

**Background:**

Digital formats have the potential to enhance accessibility to care for individuals with suicidal ideation. However, digital self-help interventions have faced limitations, including small effect sizes in reducing suicidal ideation, low adherence, and safety concerns.

**Objective:**

Therefore, we aimed to develop a remote blended cognitive behavioral therapy intervention that specifically targets suicidal ideation by blending video therapy with web-based self-help modules. The objective of this paper is to describe the collaborative development process and the resulting intervention and treatment rationale.

**Methods:**

First, we compiled intervention components from established treatment manuals designed for people with suicidal ideation or behavior, resulting in the development of 11 drafts of web-based modules. Second, we conducted a qualitative study, involving 5 licensed psychotherapists and 3 lay counselors specialized in individuals with suicidal ideation who reviewed these module drafts. Data were collected using the think-aloud method and semistructured interviews, and a qualitative content analysis was performed. The 4 a priori main categories of interest were blended care for individuals with suicidal ideation, contents of web-based modules, usability of modules, and layout. Subcategories emerged inductively from the interview transcripts. Finally, informed by previous treatment manuals and qualitative findings, we developed the remote blended treatment program.

**Results:**

The participants suggested that therapists should thoroughly prepare the web-based therapy with patients to tailor the therapy to each individual’s needs. Participants emphasized that the web-based modules should explain concepts in a simple manner, convey empathy and validation, and include reminders for the safety plan. In addition, participants highlighted the need for a simple navigation and layout. Taking these recommendations into account, we developed a fully remote blended cognitive behavioral therapy intervention comprising 12 video therapy sessions and up to 31 web-based modules. The treatment involves collaboratively developing a personalized treatment plan to address individual suicidal drivers.

**Conclusions:**

This remote treatment takes advantage of the high accessibility of digital formats while incorporating full sessions with a therapist. In a subsequent pilot trial, we will seek input from individuals with lived experience and therapists to test the feasibility of the treatment.

## Introduction

Suicide is a severe public health problem, with over 700,000 people worldwide dying by suicide each year [1]. However, suicide and suicide attempts represent only “the tip of this iceberg, which is dwarfed by the much larger problem, at least with regard to numbers, of all the people beneath the surface who are experiencing suicidal misery, often in silence” [2]. Suicidal ideation is particularly common in clinical populations; approximately 16% to 32% of patients experiencing various mental disorders report lifetime suicidal ideation [3]. Suicidal ideation is one of the strongest predictors of subsequent suicidal behavior [4,5]. The majority of transitions from suicidal ideation to suicidal behavior occur within 12 months from onset [6], and there are no robust indicators for progression to suicidal behavior [7]. Hence, timely reduction of suicidal ideation is of utmost importance.

Cognitive behavioral therapy (CBT) demonstrated effectiveness in reducing suicidal ideation and suicide attempts [8-11]. A growing body of research suggests that interventions primarily focusing on suicidal ideation or behaviors are effective in reducing these outcomes [12-15], whereas, to date, there is no robust evidence for interventions aimed at the reduction of associated psychopathology [14-18]. Unfortunately, many suicidal individuals do not receive treatment [19,20]. Barriers to treatment include a low perceived need for treatment, a preference to handle the issue independently, and stigma [19,20]. Furthermore, structural barriers such as limited time, financial resources, transportation issues, and lack of available treatment options can also hinder access to care [19,20]. Accessing specialized treatment services is particularly challenging in several countries [19]. Remote digital interventions have been developed to address these barriers.

Digital treatment formats are being discussed as a means to overcome these barriers. Research on digital interventions to reduce suicidal ideation and behaviors has predominantly focused on self-help interventions, often with little or no human support [15,21]. This approach offers high scalability, flexibility, and the potential for anonymous treatment provision [22]. Several meta-analyses have shown that digital self-help interventions can lead to significant and clinically relevant improvements in suicidal ideation [15,21,23]. However, the effect sizes are small, adherence rates are only moderate, and treatment safety remains unclear due to high dropout rates and insufficient data on suicide attempts [21]. Moreover, it remains uncertain whether self-help interventions effectively reach those who would not seek treatment otherwise, as more than 50% of participants in a meta-analytic sample were in parallel psychological or psychiatric treatment [21]. The limited effect sizes and treatment adherence in digital self-help interventions for individuals with suicidal ideation may be attributed to certain constraints inherent in current interventions.

First, all current digital interventions are self-help interventions, either with minimal or no human support. However, multiple studies have demonstrated that the adherence to and effectiveness of digital interventions can be enhanced by providing additional human support [24,25]. The level of guidance can range from brief written feedback (guided self-help [22]) to full sessions with a therapist (blended care [26]). The lack of human support is particularly concerning for the target group of individuals with suicidal ideation, as low social connectedness is considered a major risk factor for suicide in several theoretical frameworks [27,28], potentially limiting the effectiveness and acceptability of self-help interventions. Moreover, the safety of self-help interventions in this target group remains unclear [21].

Second, the majority of current digital interventions for the treatment of suicidal ideation are strictly manualized [21] and often have a strong focus on cognitive restructuring or worry scheduling [29-33]. In contrast, experts in psychological treatment for suicide emphasize the importance of individualized treatment plans according to the unique needs of each patient [34-37].

To address these limitations, we developed GO-ON*line*, a personalized blended CBT intervention for the treatment of suicidal ideation. We combined video therapy with web-based self-help modules in order to retain the advantages of a fully remote treatment while offering intensive human support. Blended care might increase the effectiveness and treatment adherence compared with digital self-help interventions [26]. In the integrated blended care model (as opposed to sequential models where the self-help modules are provided before or after face-to-face therapy), web-based modules are provided between therapy sessions and can serve as preparation or follow-up for video therapy sessions [26]. Thus, therapists can create individualized treatment plans and flexibly respond to the needs of the patient in the course of the treatment. Like digital self-help interventions, remote blended formats could enhance access to specialized care, regardless of nearby on-site treatment availability.

Despite the potential benefits, there is currently limited research on blended care [26,38]. It remains widely unclear how blended care should be designed, especially for the target group of individuals with suicidal ideation. To guide the development of our intervention, we conducted a qualitative study to investigate how blended care and web-based modules (including contents, layout, and usability) should be designed for this specific target group. This paper aims to present the development process and the resulting remote blended intervention.

## Methods

### Overview of Intervention Components and First Module Drafts

The development team had long-lasting experience both in face-to-face treatments for individuals with suicidal ideation (TT) and digital treatment formats for mental disorders (LBS). In the first step, we made an overview of intervention components used in face-to-face therapy based on CBT manuals for the treatment of suicidal ideation [34-37,39-43]. In the next step, we created a list of potential web-based modules that we considered feasible for a remote blended care intervention, and we developed first drafts for 11 web-based modules (see *Qualitative Study*, *Intervention*).

### Qualitative Study

#### Participants

We recruited licensed psychotherapists from the outpatient psychotherapy clinic of the Department of Rehabilitation Psychology and Psychotherapy, University of Freiburg. In addition, we recruited voluntary lay counselors from the Arbeitskreis Leben e.V. (AKL) Freiburg who offer companionship to individuals in crises or engage in email peer counseling for young people. AKL lay counselors often have lived experiences with suicidal ideation. Recruitment took place via flyers, personal contacts, and presentations in meetings in the respective organization. Participants were eligible if they were at least 18 years old, fluent in German, and consented to study participation. A total of 5 psychotherapists and 3 AKL lay counselors participated in the study. All therapists were females. They had a mean age of 39.8 (SD 7.3) years and 11.2 (SD 11.0) years of working experience; 4 psychotherapists reported working with patients with suicidal ideation from time to time, and 1 rarely. Two of the lay counselors were females, and 1 was male. Lay counselors had a mean age of 53.7 (SD 26.3) years and had been volunteering in the AKL for a mean of 3.7 (SD 2.5) years. One lay counselor reported working with suicidal clients from time to time, and 2 several times per week.

#### Intervention

A total of 11 module drafts were reviewed in this qualitative study. The modules delivered psychotherapeutic intervention components using text, videos, images, audios, and engaging elements (ie, questions and conditional contents in response to the entries of participants). The following module drafts were reviewed: means restriction, safety planning, reasons for living, hope box, motivational interviewing, narrative interview, chain analysis, cognitive model, dealing with perceived burdensomeness, enhancing self-esteem, and fostering social relationships.

#### Ethical Considerations

The ethics committee of the University of Freiburg approved this study (20-1349). Participants were informed about study procedures in written form and verbally and had the opportunity to ask questions. Afterward, the participants provided informed consent. The study data were anonymized, including all potential identifying data in the transcripts of qualitative interviews. Participants did not receive any compensation for study participation.

#### Procedures

The data collection took place in February 2021. After providing informed consent, participants completed a short sociodemographic questionnaire. In the second step, participants reviewed 1-3 modules each, depending on the module length. A total of 11 modules were reviewed by at least 1 participant. We investigated participants’ experience using the think-aloud method [44]: participants were asked to say anything that came to their mind while progressing through the modules. In the third step, after reviewing the modules, we conducted a semistructured interview (see Multimedia Appendix 1 for a translated version of the interview guideline). We audio-recorded the think-aloud sessions and the semistructured interviews. The interviewer took notes during the think-aloud session to document which module elements the participant was referring to.

#### Qualitative Content Analysis

The data were transcribed using f4transkript: audiotranskription. We conducted a qualitative content analysis according to Kuckartz [45] using MAXQDA 2022 (VERBI Software). We defined the main categories a priori, comprising blended care for individuals with suicidal ideation, contents of web-based modules, usability of modules, as well as layout of web-based modules. The subcategories emerged inductively from the interview transcripts. After an initial analysis of the transcripts (MN), the categories were discussed with and revised by a second researcher (RB). In addition to the general feedback analyzed in the qualitative analysis, we also extracted module-specific feedback from the transcripts (eg, feedback on a specific exercise used). However, to make this paper more accessible to a broader readership, this report focused on the general findings on blended care for people with suicidal ideation.

### Design of the Blended Intervention

We revised the drafts of the web-based modules and developed additional modules based on the feedback from therapists and lay counselors. In addition, we developed a treatment manual to guide the therapeutic process. The development team reviewed and discussed the recommendations and incorporated them into the design of web-based modules and the treatment manual when deemed reasonable and applicable.

## Results

### Qualitative Findings

A brief overview of the qualitative results is provided here; see Multimedia Appendix 1 for the detailed findings along with quotes from psychotherapists and lay counselors.

#### Perspectives on Blended Care for People With Suicidal Ideation

Therapists and lay counselors expressed that a blended approach would enrich therapy and strengthen the autonomy and self-efficacy of patients. They found it beneficial that modules could be used to outsource content, such as psychoeducation, and bridge the time between sessions by guiding patients through the topics and providing the option to review content. The participants emphasized the role of the therapist in helping patients through problems and encouraging them, especially since some modules can be burdensome for this target group.

Participants offered recommendations for the design of a blended care intervention, suggesting that therapists should thoroughly prepare for web-based therapy with the patients. Data security and privacy should be ensured, and patients should be informed who reads their entries and how they will be used in therapy (eg, to prevent fear of involuntary hospital admission). Additionally, participants recommended considering the suitability of web-based therapy for each individual patient, taking into account their personal preferences, computer skills, and intellectual abilities. It was highlighted that the therapy should be adapted to the specific needs of the individual patient, for example, aligning with different mental disorders or severity of suicidal ideation. The participants named several important topics and components in therapy for individuals with suicidal ideation, for example, reasons for living, perceived burdensomeness, and risk assessments.

#### Contents of Web-Based Modules

Participants recommended keeping the explanation of concepts simple and brief. Furthermore, modules should use a nonacademic language, especially due to frequent concentration difficulties in this target group. The benefit of example patients was discussed; participants suggested that they should be similar to the patients (eg, in terms of age and life situation) and described with sufficient detail. The modules should transmit empathy and validation, for example, by responding to the patients’ entries, validating their experiences, and encouraging them to reward themselves. Participants recommended that modules should contain engaging exercises. The therapists and lay counselors discussed how safety strategies can be implemented in web-based modules: this could include reminders for the safety plan and emergency numbers. In addition, they suggested compiling possibilities to distract or reward themselves before working on the modules.

#### Navigation

Participants often noted that a simple navigation through the web-based modules is required. Instructions might facilitate navigation where necessary. In addition, participants suggested using a general introduction to the therapy and an overview of the web-based modules.

#### Layout

The participants stated that modules should have a clear, aesthetic, and simple layout. The layout should support the contents, for example, by highlighting important elements. Participants recommended using pictures that match the patients’ reality and have a clear connection to the module contents.

### Further Development of the Blended Intervention

We revised the modules and designed the treatment manual in line with the feedback from therapists and lay counselors. Furthermore, we developed a 2-day training to instruct therapists for treatment. The training included 1 day of training on face-to-face interventions for patients with suicidal ideation as displayed in the manual, and 1 day of training on the use of the web-based modules and particularities of video therapy.

Meeting the recommendations for blended care design, we specified how to blend the modules with the therapy sessions, including hints for potentially burdening modules to specifically prepare the patients for the therapy sessions. We developed an informed consent sheet detailing the safety procedures and we added information on data privacy for the patients to an introductory web-based module. Following the recommendation to adapt the treatment to the needs of the respective patient, we developed an individualized treatment format. We incorporated the treatment components suggested by participants.

Concerning the contents of web-based modules, we addressed the recommendations by simplifying the language and explanations of concepts and adding graphics where applicable. Furthermore, in an attempt to provide patient examples with sufficient similarity to each patient, we chose 3 example patients who covered different psychopathogeneses, genders, and age groups. Furthermore, we incorporated the feedback on how to transmit empathy and validation in web-based modules. We addressed the safety recommendations by adding reminders about the safety plan and the emergency number to each module. In addition, we discussed and addressed any module-specific feedback.

Regarding the navigation and layout of modules, we standardized the module procedures based on the feedback. This included an overview of the module at the beginning and an outlook on the next steps at the end of each module. We developed an introductory module that provided general information about the web-based modules and the therapy process. For patients with lower digital literacy, we provided a sheet explaining the login and registration process. We developed a simple and consistent module layout in line with participants’ feedback. Moreover, we inserted pictures with a clear connection to the contents and to the everyday lives of patients.

### Resulting Blended Care Intervention

The final GO-ON*line* program was designed as a remote blended intervention, consisting of 12 sessions of outpatient psychotherapy via a videoconferencing system and web-based self-help modules. The manualized program is based on CBT and strictly focuses on the treatment of suicidal ideation and behaviors, as proposed in previous manuals on the treatment of patients with suicidal ideation [34-37,39-43].

**Figure 1:**
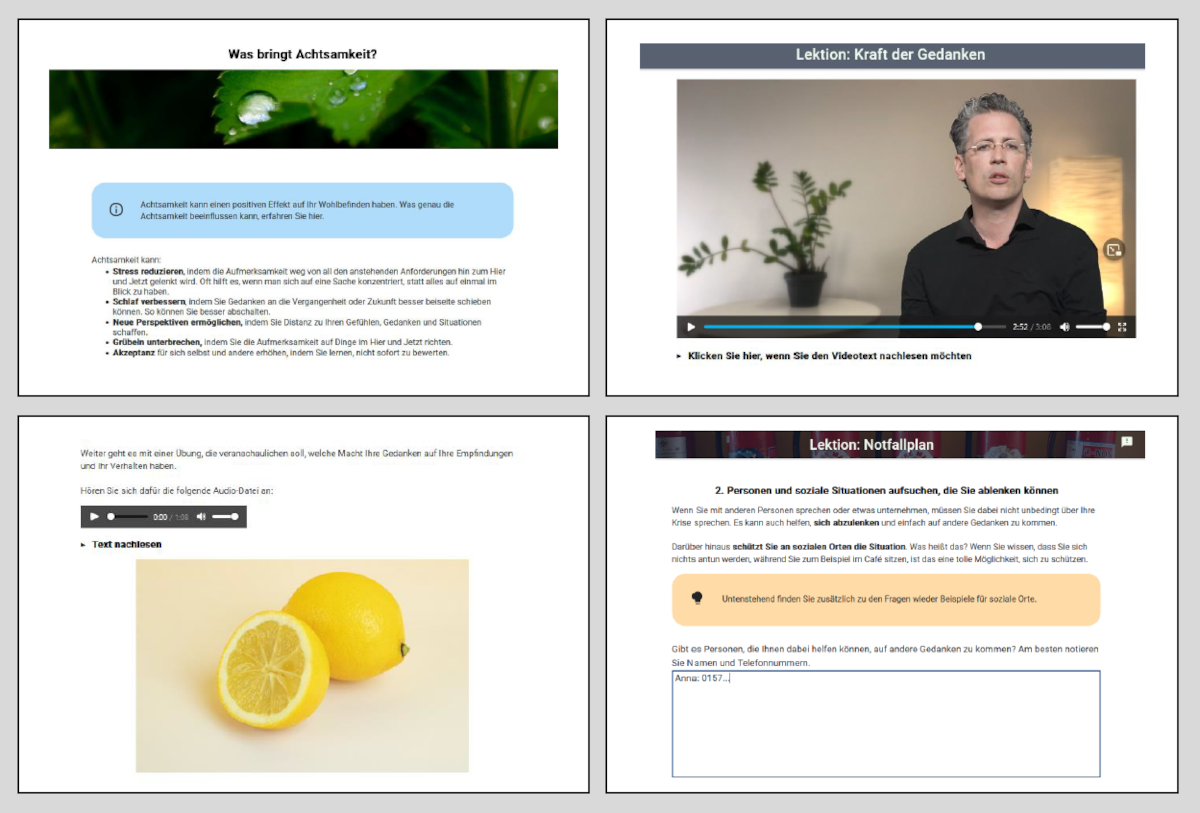
Screenshots from web-based modules (ie, psychoeducational text and video, audio exercise, and development of the safety plan).

We developed 31 web-based modules covering a wide range of intervention components; a full list is displayed in Table 1. We incorporated most components we identified in CBT treatment manuals into the intervention, with few exceptions (eg, when there was mixed evidence such as for antisuicide contracts [46], or when the intervention seemed too complex for a short-term therapy). In the blended intervention, there are no obligatory web-based modules: any relevant modules can be selected and assigned based on the individual case conceptualization and treatment plan. Patients are supposed to work on the modules between the video therapy sessions as a preparation or follow-up. The therapist can view the patient's entries in the web-based modules. Example screenshots are displayed in Figure 1.

**Figure 2:**
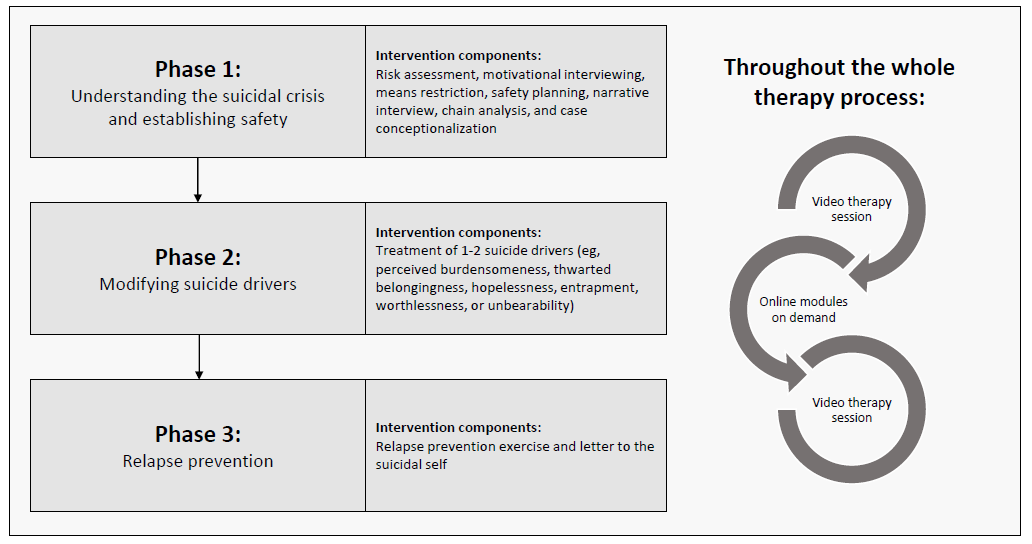
Blended intervention design. The treatment consists of 12 sessions of video therapy combined with web-based modules that are selected based on the individual treatment plan. All components of the first treatment phase need to be completed in order to establish safety; in treatment phase 2, individual suicidal drivers are targeted. There are no obligatory web-based modules.

The treatment process is divided into 3 phases (compare [37]; Figure 2): during the initial phase, the objectives include understanding the suicidal crisis, conducting a risk assessment, identifying reasons for living, and implementing crisis therapy strategies. These strategies encompass the development of a safety plan, means restriction, and the establishment of strategies to address symptom load (eg, panic, nightmares, insomnia, dissociation, and rumination). At the end of the initial phase, an individual case concept is developed and discussed with the patient. The case concept guides the intervention selection in the second treatment phase. Accordingly, the focus of the second treatment phase centers on addressing 1 or 2 core “suicidal drivers” [35], that is perceived burdensomeness, thwarted belongingness, hopelessness, entrapment, worthlessness, or unbearability (Figure 2). Standard cognitive behavioral therapy methods (eg, cognitive restructuring, problem-solving, and behavioral activation) are used to address the different “suicidal drivers.” In the final phase, relapse prevention strategies (relapse prevention task and letter to the suicidal self) are implemented to prepare patients for potential future suicidal crises. All treatment components of the initial therapy phase are obligatory, whereas all further treatment modules are freely selectable based on the individual case conceptualization.

The treatment manual includes the following safety protocol. First, at the beginning of each video therapy session, therapists perform a risk assessment. Second, patients are informed that module contents and daily diary are not reviewed outside of the therapy sessions. Third, patients are required to consent to perform therapy sessions from the same address. In cases where a patient fails to attend a scheduled therapy session and cannot be reached within 15 minutes, a designated contact person is immediately called. If deemed necessary, therapists call the police.

**Table 1:** Overview of web-based modules.

Number	Module title	Description
1	Introduction	Introduction to web-based modules and overview of psychotherapy program
2	Treatment log	Overview and description of all available web-based modules
3	Strengths and resources	Psychoeducation and questionnaires on strengths and resources
4	Reasons for living	Reasons for living questionnaire
5	Motivational interviewing	Collecting personal motivations for dying, against dying, and for living
6	Diary card	Introduction to the daily symptom diary
7	Narrative interview	Writing down the personal history with suicide
8	Chain analysis	Detailed analysis of the short-term events that led up to a suicidal crisis (suicide attempt or ideation)
9	Safety plan	Development of a personal safety plan and identification of warning signs
10	Means restriction	Psychoeducation and options for means restriction
11	Perceived burdensomeness	Cognitive restructuring: perceived burdensomeness
12	Thwarted belongingness: reviewing the personal network	Writing down the personal network and star exercise
13	Thwarted belongingness: establishing new contacts	List of options where new contacts can be established
14	Hopelessness	Cognitive restructuring: hopelessness
15	Skills	Psychoeducation and list of dialectical behavior therapy skills
16	Problem-solving	Problem-solving scheme
17	Self-esteem	Cognitive restructuring: low self-worth
18	Cognitive model	Psychoeducation on cognitive model
19	Cognitive restructuring	Cognitive restructuring worksheet
20	Hope box	Collecting hope-inspiring objects, quotes, and symbols in a box
21	Symptom control: dissociation	Psychoeducation on dissociation
22	Symptom control: hyperventilation	Psychoeducation on hyperventilation and breathing exercise
23	Symptom control: rumination	Psychoeducation on rumination, rule for detecting rumination, reducing triggers for rumination, list of strategies (distraction, activities, or sensory impressions), worry scheduling, and cognitive restructuring on potential promises of rumination
24	Symptom control: sleep problems	Sleeping rules
25	Mindfulness	Psychoeducation on mindfulness and mindfulness exercises
26	Progressive muscle relaxation	Psychoeducation on progressive muscle relaxation and exercises
27	Behavioral activation	Psychoeducation on behavioral activation and identification of vital and meaningful activities
28	Positive psychology	Psychoeducation on happiness, gratitude journal, and end-of-day review
29	Letter to the suicidal self	Writing a letter to the future self, reflecting on helpful strategies, and cognitions from the therapy
30	Reports from suicide survivors	Collection of written reports from suicide survivors
31	Emergency materials	Emergency numbers and personal safety plan

## Discussion

### Principal Findings

In this study, we have developed a remote blended treatment for individuals with suicidal ideation, integrating video therapy and digital self-help modules. Qualitative interviews with therapists and lay counselors offered valuable insights on how to design blended care specifically for this target group, with web-based modules being recognized as a valuable complement to therapy sessions. Intervention components were selected based on previous treatment manuals for this target group [34-37,39-43]. The remote blended format is designed to leverage the high accessibility of digital formats while incorporating full sessions with a therapist.

In the treatment design, we carefully addressed several important issues that emerged from this qualitative study and the review of previous treatment manuals. In terms of treatment safety, therapists and lay counselors highlighted that certain web-based modules, such as motivational interviewing or narrative interviews, could be particularly challenging for patients as they confront them with a suicidal crisis. They recommended to carefully integrate the self-help modules into therapy sessions, enabling therapist to address potential issues and unwanted effects. Therefore, we flagged potentially burdensome web-based modules in the manual and provided guidance on how to prepare for and follow up on critical modules during the therapy sessions. Importantly, all therapy programs that have demonstrated efficacy in reducing suicidal behaviors [34,37,47] include specific strategies to respond to suicidal urges [48]. Based on these treatment manuals, we prioritized treatment safety as the first and only obligatory treatment target. Safety planning [49] and means restriction [50] have been shown to be effective in preventing suicidal behavior and were therefore implemented in the program. In line with the feedback from participants, the web-based modules include regular reminders about the safety plan, taking a break, or calling an emergency number if necessary.

Another issue to consider in blended care for individuals with suicidal ideation is the high heterogeneity in mental disorders or life circumstances associated with a suicidal crisis, as well as varying levels of severity of suicidal ideation and behaviors. The therapists and lay counselors emphasized the importance of tailoring the intervention to meet each patient's specific needs, highlighting the need for a personalized treatment approach. In addition to increasing the acceptability [51], personalized interventions aim to increase the effectiveness of interventions [52]. In our program, therapists create an individual case conceptualization and adapt the therapy to suit the needs of each patient. The treatment manual offers a range of optional intervention components to target individual suicidal drivers [35]. However, developing web-based modules for this broad target group is challenging. To address this, we provide a large pool of 31 modules, from which only the relevant ones are selected in a shared decision-making process. Additionally, we have included patient examples representing various clinical and sociodemographic characteristics.

Based on the participants’ feedback, we designed the web-based modules as simple and user-friendly as possible, considering that many patients struggle with concentration issues.

Several limitations need to be considered. First, we did not include participants with lived experience of suicidal ideation. A participatory approach involving people with lived experience is strongly recommended [53]. However, this paper represents the initial step in treatment development; input from people with lived experience of suicidal ideation will inform the further revision of the intervention as part of a feasibility trial. Second, the sample size in the qualitative study was small, and we did not assess whether data saturation was reached. Obtaining more feedback is necessary to ensure a comprehensive understanding. To address this limitation, we are planning a larger study with a more diverse participant pool to capture a broader range of perspectives and experiences. Third, the therapists’ views might partly reflect their knowledge of and attitude toward digital interventions. Therapists had moderate experience with treating patients with suicidal ideation and were recruited from a department where digital interventions were a primary research focus, potentially limiting the range of responses. Moreover, participants’ perspectives might be partly biased by common assumptions or personal experiences, such as their expectations regarding the age group that would benefit from blended care. Thus, we will capture the actual experiences of therapists treating patients using a blended care program in an upcoming feasibility study. Fourth, the therapists and lay counselors did not review the treatment manual during its development, as it was created after conducting the qualitative interviews. Nevertheless, the insights gained from these interviews played a crucial role in shaping the entire treatment program. The manual itself is based on previous face-to-face treatment manuals, and as part of the feasibility study, we will seek feedback to further refine it.

### Conclusions

In conclusion, our remote blended care treatment, which integrates video therapy and digital self-help modules, holds promise in meeting the needs of individuals with suicidal ideation. The program prioritizes safety planning and means restriction as essential treatment components while tailoring the treatment to the specific needs of each patient. Further research, including the perspectives of people with lived experience, is essential to determine the intervention’s effectiveness, acceptability, and practicality in comparison to established care models.

## Appendix

Multimedia Appendix 1 Semistructured interview guideline and detailed qualitative findings.

## Abbreviations

AKL: Arbeitskreis Leben e.V.
CBT: cognitive behavioral therapy

## Data Availability

The data sets generated and analyzed during this study are not available due to informed consent. The treatment manual and access to the web-based modules are available from the corresponding author on reasonable request.
