# Supplementary material for: Blending Video Therapy and Digital Self-Help for Individuals With Suicidal Ideation: Intervention Design and a Qualitative Study Within the Development Process
Source: JMIR Form Res. 2023 Sep 21;7:e49043. doi: 10.2196/49043 (PMC10557000; doi:10.2196/49043)
Supplement: Multimedia Appendix 1 [file formative_v7i1e49043_app1.doc]

Semi-structured interview guideline:

|  | **Questions** | **Additional questions** |
| --- | --- | --- |
| Introduction | What are your thoughts on the modules?  Please take your time to tell me what you noticed while going through it or what you think about the online modules. Feel free to tell anything that comes to mind. I will not interrupt you for now. | Is there anything else you would like to comment on? |
| Concept | Do you think the online modules are appropriate for therapy-accompanying treatment of suicidal ideation?   - What should be considered in the process? - What challenges do you see in the use of online modules with people with suicidal ideation?   From your point of view, what should be considered in the treatment of people with suicidal ideation?  - - Can this be realized in online modules?  Do you see advantages and opportunities in the use of such online modules next to therapy therapy sessions?  - What would this change? | Why/Why not?  Anything else?  Why?  How?  Which ones? |
| Content | How appropriate are the content of the modules for patients with suicidal ideation?   - Are there any contents that should definitely be changed? - What should be improved? | Which? How? |
| Design | What did you think of the design and technical implementation of the modules?   - Do you think patients can navigate the different modules well? - Do you see any difficulties using the modules? - Which modules could be improved? | How? |

**Detailed qualitative findings:**

***Views on blended care for individuals with suicidal ideation***

The therapists and lay counsellors indicated that a blended concept “*can enrich the therapy” (T948)*. They highlighted several advantages of blended care, including the following aspects of online modules: It was often noted that online modules strengthen the autonomy and *“this unconsciously promotes a lot of self-efficacy” (T736).* Two participants noted that the modules are highly flexible, and three stated that patients can use them as a tool to observe changes. Most participants emphasized that modules can outsource therapy contents*.* Furthermore, four participants found that the modules can bridge the time between therapy sessions: *“What is actually happening between one and the other therapy session? […] This is a lot of time. I think that this is an opportunity to continue the therapy even though there is nobody who listens” (C205)*. Some participants also noted that modules can guide patients through therapeutic topics, that modules provide repetition and memory support, and that patients have the possibility to review the contents. Two participants mentioned the low-threshold nature of online therapy.

In addition, the participants found that the possibility to discuss the modules in a therapy session very helpful. They often emphasized that aspect of encouraging patients and helping them through problems: *“when you have somebody in front of you, you notice that, you feel that, and can rephrase this. And if it is only online, you will not notice who drops out. And this could be a problem.” (T309)* Three therapists indicated that the modules can be temporarily burdensome and patients might be overstrained with some tasks. Two participants also noted that patients might not engage with modules properly.

Participants named several preconditions for blended care. One therapist highlighted the need to prepare online therapy with patients *“because this is very challenging and you have to focus and I think this is cognitively and emotionally very burdening […] And this is why I think it is important that there is some kind of a preliminary meeting” (T736)*. One therapist noted that they would *“only activate the modules that are actually the subject of therapy right now, because [….] patients tend to look at this and that […] and then there are things that stir them up” (T217)*. Furthermore, three participants stated that the data security and privacy should be given priority, and that patients need to know that their entries will not be used against them: *“But at the same time, there could be the fear: Is this a surveillance measure? And is this being evaluated online and are the data forwarded, so that it can get to a hospital admission against my will?” (T217)*.

In addition, the participants discussed the target group. Three participants said that the fit of a patient with online therapy should be clarified; three participants stated that it could be appropriate for people who are looking for online support (or open to it). Four participants said that low computer skills could be hindering; it was also noted that young people might be more open to online therapy. One lay counsellor said that the online modules required some degree of intellectual abilities. Several therapists highlighted that the therapy should be adapted for people with different disorders or backgrounds, or with a different severity of suicidal ideation.

The participants named several important topics in therapy for individuals with suicidal ideation. This included reasons for living, perceived burdensomeness, and behavioral activation. Furthermore, there should be risk assessments, the topic of asking others for help, de-stigmatization of suicidal ideation, and the notion that crises pass. Three participants stated that therapy or self-help modules in general should build a bridge to other services*.* One lay counsellor said that suicide risk should be taken seriously: *“You must not trifle with this in any case. Even if you personally have the feeling that he is not so much at risk” (C386)*.

***Contents of online modules***

Several participants thought that the explanation of concepts should be simple and brief: *“And I find it a bit too, how should I say this… Somewhat overly theoretical, overly academic I say. The things being said are correct. But I am asking myself whether this actually gets through to someone in a crisis situation?” (C205).* Many participants emphasized the need for a simple and non-academic language. Two therapists noted that many patients have concentration difficulties. Furthermore, two therapists stated that repetition in modules should be avoided. Four therapists found that examples should be used to illustrate concepts or tasks: *“Anyhow, I like the examples for warning signs. […] So that patients have the opportunity, okay, one or two things I already know about me, but when they read examples, they come up with more.” (T736)* In addition, the benefit of example patients was noted. Four participants highlighted that example patients should be similar to the patients: *“When I think that this is for people from 18, then I find the examples… somehow insufficient, because they are about caring about kids, going to work. And I would like an example for the younger target group, don’t know, around 20, going to university, so that this is more accessible” (C278).* One therapist found that the example patients should be provided with sufficient detail: *“Is he actually alone? Does he have people around himself?” (T217).* In addition, two therapists suggested that graphics should be used to convey the contents.

The participants discussed how modules can transmit empathy and validation for patients; two therapists noted that *“considering the fact that you sit alone in front of a computer, you still have the feeling that you are being cared for.” (T736).* Four therapists highlighted that it is helpful if modules encourage patients to reward themselves or to relax. Furthermore, they noted that modules should respond to the patients’ entries: *“I liked that the module responds to the mood that I indicate, so it does not always respond the same when I feel bad, good, neutral, or really bad.” (C205)*. Most participants emphasized the importance of validating the patients in the modules: *“What I also really liked was these texts and great, you made it, and you can be proud of yourself” (C278)*. Three participants stated that the modules should be designed personal and approachable where possible; one lay counsellor suggested to address patients informally.

Several participants said that interactive exercises should be included; two participants said that the questions in online modules should be clear and precise. In addition, several participants stated it is helpful to have subtitles in videos or the videotext below.

The participants discussed safety strategies that can be implemented in online modules. Two participants each pointed out the need for safety planning and for emergency numbers in the modules. Four participants said that modules should include reminders for breaks or the safety plan: *“I think these are really strong emotions and… Would it maybe be good if there was a hint: If you notice that this stirs something up, please take a break and look at your safety plan. I think exactly this part will trigger a lot in this module” (T736).* Two therapists highlighted the importance of collecting good resources to distract or reward themselves before working on the modules: *“Caution! Big problem. The patient is now really downcast, and in this state, he might not come up with anything to reward himself […] This is why it is very important that before an exercise or a module, they think about what to do afterwards. That they plan something, if possible not alone, going outside, doing something else” (T217).*

***Usability of online modules***

It was often noted that a simple navigation is required. The participants often commented on navigation issues while they were working on the modules, found something complicated, or did not know where to continue: *“Okay, so I can’t get further, when I click here, it does not do anything” (C386).* Two participants suggested that it can sometimes be helpful to repeat the patients’ entries later in a module; two therapists found a progress bar helpful. In addition, the potential use of instructions was discussed. Five participants suggested instructions could improve the navigation through modules: *“Should there maybe be a hint or something like a guideline how to move through the modules?” (C205)*. Nevertheless, two therapists found that instructions are not necessary if the navigation is simple: *“I suspect that the patients who use this won’t have any technical problems with it. Even if they have them, they can solve them by themselves in this moment […]. And conversely, patients who have difficulties to understand what to do, and what is scrolling and what is clicking and so on, they won’t do it at all.” (T217).* Furthermore, five participants suggested to provide guidance for module contents and tasks: *“Yes, information always before the task. This simplifies it, I think, because people like me would not start directly doing this right away, and then feel overstrained, not knowing what to type in there.” (T948)*. Finally, three participants stated that a general introduction would be helpful, guiding through the whole therapy and providing an overview of the online modules.

***Layout of online modules***

The participants stated that there should be a clear, aesthetic, and simple layout with an appropriate color scheme, consistent within and across modules. Two therapists noted that the layout should support the contents. Four participants stated that important elements should be highlighted. Five participants stated that elements and fonts have an appropriate size: *“The logo of the study is very big. But the button to play the video is somehow a bit small” (T217)*. Three participants stated that it was good to have pictures in the modules. However, it was often noted that pictures should have a clear connection to the module content. One therapist emphasized that the pictures should match the patients’ reality: *“Again, this is a somewhat euphemistic image of someone walking towards the sunset with a kite. I think that’s just pretty far away from real life” (T620).* Three therapists found that pictures should be motivating or hope-inspiring.
